# Leveraging multimodal machine learning for accurate risk identification of intimate partner violence

**DOI:** 10.1038/s44294-025-00126-3

**Published:** 2026-03-13

**Authors:** Jiayi Gu, Kimberly Villalobos Carballo, Yu Ma, Dimitris Bertsimas, Bharti Khurana

**Affiliations:** 1 Trauma Imaging Research and Innovation Center, Brigham and Women’s Hospital, Boston, MA USA; 2 Operations Research Center, Massachusetts Institute of Technology (MIT), Cambridge, MA USA; 3 Sloan School of Management, MIT, Cambridge, MA USA; 4 Dynamic Ideas LLC, Waltham, MA USA; 5 Harvard Medical School, Boston, MA USA

**Keywords:** Diagnosis, Health services

## Abstract

Intimate partner violence (IPV) refers to the abuse from previous or current partners. It is a widespread but underreported public health concern that has a wide range of negative effects on the physical and mental health of those affected. This work presents machine learning models for the early detection of IPV in clinical settings, developed with a dataset of female patients who sought help at a domestic abuse intervention and prevention center of a major hospital in the United States. Utilizing tabular clinical data and unstructured clinical notes, we build single-modality and multimodal models for different data availability scenarios. Our multimodal model can identify patients at risk of IPV with an AUC of 0.88 and years before patients seek help. We validated the model on patients who did not seek help at the intervention center and patients from another hospital in the same integrated network with comparable performance.

## Introduction

Intimate partner violence, or IPV, is characterized by violence or aggression by current or former spouses and partners^1^. IPV is a significant public health challenge that affects people from all socioeconomic and geographic backgrounds. The World Health Organization classified IPV as the most widespread form of aggression against women worldwide. According to the Centers for Disease Control and Prevention, more than one-third of women in the United States will experience IPV at some point in their lives^1^. In addition to potentially life-threatening injuries, IPV is associated with negative physical health outcomes such as chronic pain, sexually transmitted infections, menopausal symptoms, and mental health disorders such as depression, PTSD, anxiety, and self-harm^2–4^. Early detection and intervention of IPV are crucial in preventing the worsening of health in those affected. However, victims may hesitate to disclose abusive relationships due to factors such as safety concerns and financial dependency on the abuser^4^, presenting challenges for timely intervention.

Healthcare clinicians have a unique edge in identifying early signs of IPV, as victims present to them frequently before they seek help from law enforcement or social agencies. The United States Preventive Services Task Force recommends routine screening for IPV in all women of childbearing age in healthcare settings^5^. However, the current screening tools capture only a fraction of cases as they simply rely on self-reporting of violence. Given the hesitancy to seek help by IPV victims due to fear, stigma, and safety concerns, many cases go undiagnosed, leading to missed opportunities for timely intervention. As a result, there is an unmet critical medical and social need for a more objective and comprehensive screening method to detect IPV.

Recent studies have demonstrated that historical clinical and imaging studies can provide valuable information in detecting IPV risk^6–10^. In particular, with access to the imaging history of patients, radiologists have an advantage in recognizing signs of IPV. Patterns in radiology studies, including high frequency of radiology imaging and injuries to the face, neck, and upper extremities, can indicate the likelihood of IPV^11–16^. However, time constraints, silos of subspecialization, cognitive overload, and the urgency to address immediate symptoms often limit the ability of radiologists and clinicians to leverage this information^8^. This motivates the need for an automated decision support tool that can utilize radiology findings and other clinical information to improve IPV identification, as prior work has shown that incorporating clinical information helps improve the accuracy of radiology interpretation and reporting^17^. Such a tool can help healthcare providers identify those at risk of IPV, allowing them to connect patients to appropriate medical and social resources effectively. There exists prior work on building machine learning tools for IPV identification^18–20^. However, they focused on relatively small populations or on predicting intervention response rather than broadly identifying risk in clinical settings.

Recognizing this potential, our work introduces the first multimodal machine-learning approach that incorporates clinical notes (including radiological notes) and structured data from Electronic Medical Records (EMR) to identify patients at risk of IPV in clinical settings. Recent advances in off-the-shelf large language models (LLMs) pre-trained on large corpora of medical texts have demonstrated strong performance in medical language learning tasks^21–24^, allowing for an effective framework for learning from unstructured texts such as radiology notes^6^. Moreover, multimodal machine learning frameworks have been shown to offer superior performance in clinical tasks compared with single-modality approaches. Soenksen et al. proposed a Holistic AI in Medicine (HAIM) framework that utilizes pre-trained feature extractors (such as medical LLMs) to unify learning from different data modalities, including tabular data, language, and imaging data. This multimodal approach consistently outperforms single-modality models for various clinical prediction and diagnostic tasks^25^.

In this work, we propose three distinct models of IPV risk detection: two single modality models utilizing tabular data or clinical notes, and a Holistic AI in Medicine (HAIM) multimodal fusion model. These models are developed with a dataset of female patients enrolled in a domestic abuse intervention and prevention (DAIP) center (cases) at a U.S. academic health center (AHC1), along with age and demographics-matched control patients from the same AHC. The different models are designed to accommodate different clinical data availability scenarios, ranging from settings with clinical note data to those with only tabular data. We evaluate and validate the performance of the models to show their potential for real-world clinical application. Our results show that the multimodal fusion model offers the strongest and most stable performance.

## Results

### Experiment setup

The IPV group used to develop the models consists of 841 female patients enrolled in the domestic abuse intervention and prevention center at a US Academic Health Center (AHC1) between 2017 to 2019 and 2021 to 2022, through either self-reporting or clinician referral. We exclude patients from 2020 due to its unique nature because of COVID-19, marked by a drop in patients reporting IPV and notable shifts in injury patterns^26^. This group is accompanied by 5,212 demographics-matched non-IPV (control) patients. The control group is constructed in a two-step process: (1) random sampling of female patients in the AHC, excluding any patients who carry any ICD code diagnoses of physical and non-physical abuse (2) matching based on demographic characteristics to ensure similarity to the IPV group. Details on the matching process are provided in the Methods section.

**Fig. 1:**
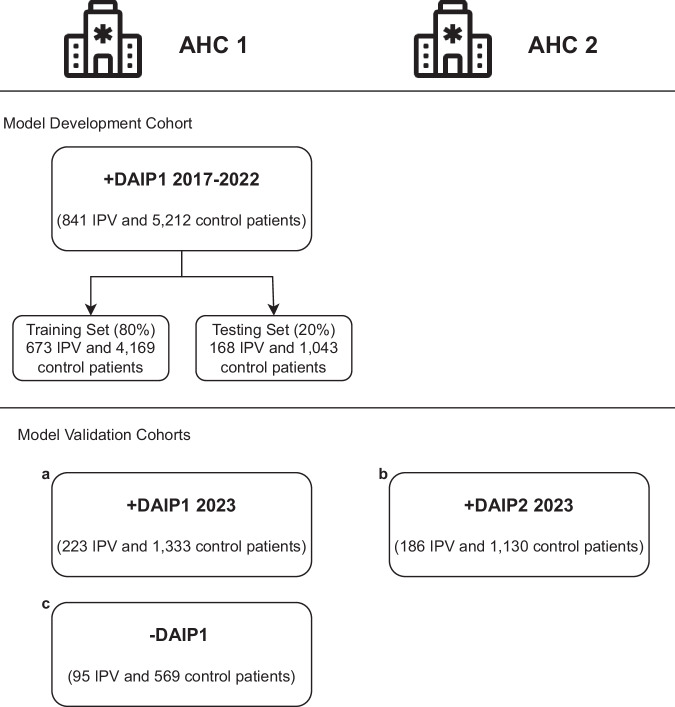
Model development and validation cohorts. The patients come from two U.S. Academic Health Centers (AHC1 and AHC2) in an integrated care system. The models are developed with patients enrolled in the domestic abuse intervention and prevention center in AHC1 from 2017-2022 (AHC1 + DAIP1 2017-2022), excluding 2020 patients. The patients are separated patient-wise into training (80%) and testing (20%) sets. The models are validated on three cohorts of patients: (**a**) AHC1 + DAIP1 2023: patients enrolled in DAIP1 at AHC1 during 2023, with optimally-matched control patients, (**b**) AHC2 + DAIP2 2023: patients enrolled in DAIP2 at AHC2 during 2023, with optimally matched control patients, (**c**) AHC1-DAIP1: patients with IPV-related diagnoses but did not seek help from DAIP1 at AHC1 from 2021 to 2023, with optimally-matched control-patients.

We validate our models on three cohorts of patients, each with demographics-matched control patients. The IPV patients in the first validation cohort include AHC1 patients who enrolled in the DAIP in 2023. Recognizing the limitation of using patients motivated to seek help at the DAIP centers, the second validation cohort includes patients who carried diagnoses of IPV at the AHC1 from 2021 to 2023 but did not enroll in any DAIP. This group of IPV patients is defined as carrying ICD-10 code diagnoses that include both physical and non-physical abuse. The last validation cohort includes IPV patients enrolled in DAIP2 in AHC2, a different hospital from the same healthcare network. Patients under 18 were excluded from all cohorts. Figure 1 summarises all cohorts for model development and validation.

To assess each patient’s evolving risk of IPV, we make daily predictions when patients encounter the hospital system on whether they might be experiencing IPV. To prevent target leakage, we adopt a patient-level splitting strategy; we train with 80% of the patients and test with 20% of the patients. All observations of a specific patient are assigned to either the training or testing group. For self-reported IPV patients, all corresponding observations are labeled as ‘IPV’ (case), while all observations for control patients are labeled as ‘non-IPV’ (control). This labeling scheme maintains consistency in classifying IPV observations, encouraging the model to capture risk factors that could indicate a higher likelihood of IPV, detect early signs of abuse before self-reporting, and allow for continuous monitoring of IPV likelihood over time.

### Model performance

We evaluate model performance using the area under the receiver operating characteristic curve (AUC), sensitivity (true positive rate), and specificity (true negative rate), which are well-suited metrics for evaluating unbalanced learning taks^27^. For IPV identification in practice, it is crucial to balance sensitivity and specificity. False negatives can lead to victims not receiving care or intervention, leaving them vulnerable to continued abuse. False positives can result in individuals being wrongly labeled as victims, leading to unnecessary interventions, emotional distress, and possible harm to the patient-clinician relationship. We report these values for both the test set and the validation sets.

**Fig. 2:**
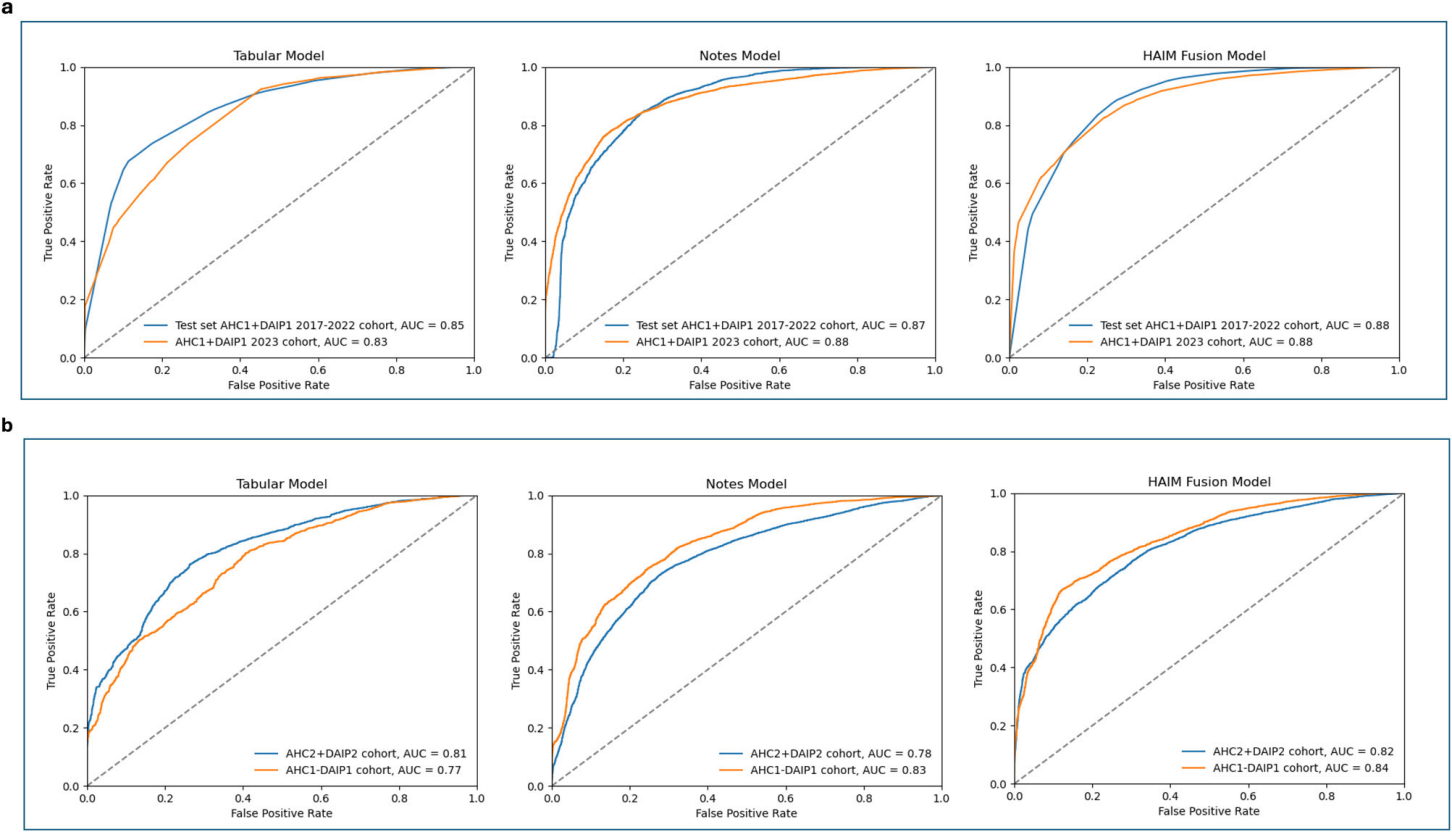
The receiver operating characteristic (ROC) curves of the tabular model, notes model, and HAIM fusion model on all cohorts. **a** On the out-of-sample test set in the model development cohort (AHC1 + DAIP1 2017–2022 cohort), the tabular model has an AUC of 0.85 and the notes model has an AUC of 0.87. The HAIM outperforms both single-modality models with an AUC of 0.88. On the internal validation cohort (AHC1 + DAIP1 2023 cohort), all models maintain an AUC above 0.83, with the HAIM fusion model having a nearly equal AUC of 0.88. **b** On the IPV patients in AHC2 (AHC2 + DAIP2 2023 cohort), the tabular model has an AUC of 0.81 and the notes model has an AUC of 0.78. On the AHC1 IPV patients who did not seek help at the domestic abuse intervention and prevention center, the tabular model has an AUC of 0.81 and the notes model has an AUC of 0.78. The HAIM fusion model maintains stable and superior performance, with AUCs of 0.82 and 0.84 for the two cohorts respectively. AHC=Academic Health Center, DAIP=domestic abuse intervention and prevention.

On the out-of-sample test set in the AHC1 + DAIP1 2017–2022 cohort, the tabular models can predict IPV risk with an AUC of 0.85 and the clinical notes model with an AUC of 0.87. The HAIM fusion model yields an AUC of 0.88, outperforming both single-modality models. On the AHC1 + DAIP1 2023 cohort, all models maintain an AUC above 0.82, with the HAIM fusion model having a nearly equal AUC of 0.88. Although the HAIM fusion model consistently outperforms the single-modality models, the tabular data-only model can perform comparably as a standalone model for IPV risk assessment when clinical texts are unavailable or difficult to collect. The performance of the three models on the AHC1 + DAIP1 cohorts is shown in Fig. 2a. The AUC performance of the tabular and fusion model across demographic subgroups is presented in Supplementary Tables 1A and 1B.

We calibrate the predicted probabilities to accurately reflect the likelihood of IPV presence using isotonic regression^28^ (results in Supplementary Fig. 1). Given the calibrated probabilities, we evaluate the Tabular and Fusion models’ accuracy, sensitivity, and specificity. We compare and select the probability threshold that balances test-set sensitivity and specificity. The comparison and the performance of these thresholds on the internal validation cohort are presented in Supplementary Table 2. Both models can achieve above 73% sensitivity and specificity with the chosen threshold.

### Prediction-report lead time in self-report patients

**Fig. 3:**
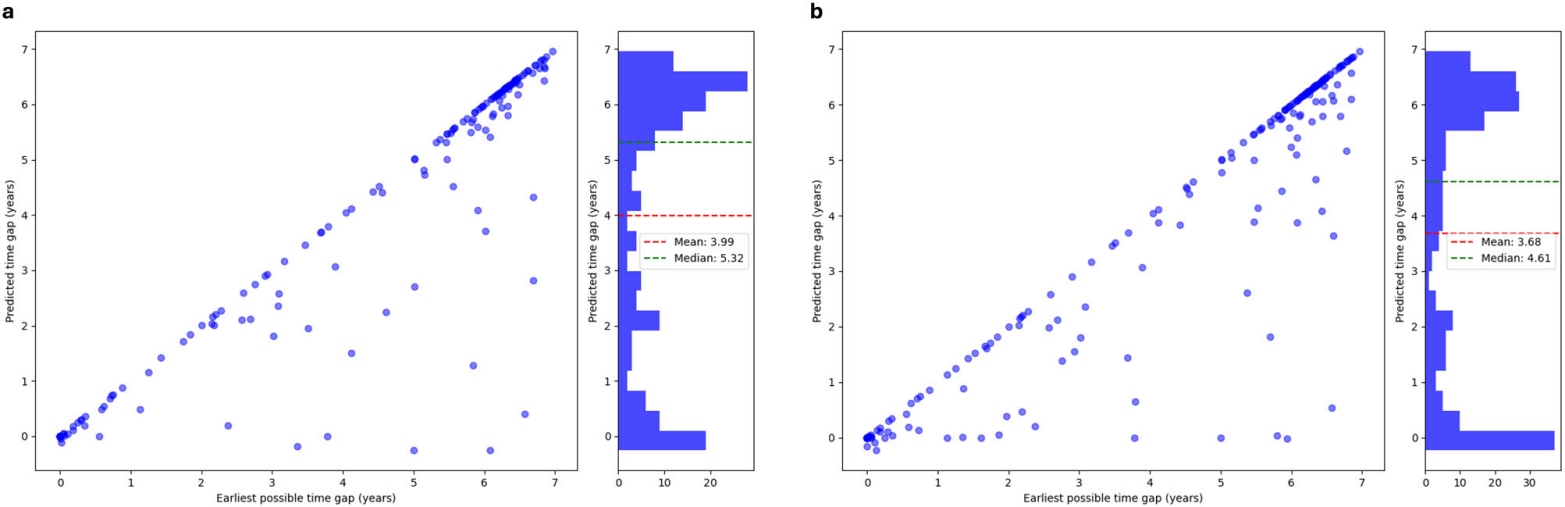
Prediction-report lead time in self-report patients. The prediction-report lead time is calculated as the difference between the patient’s first self-report date and the earliest date when the model detected IPV; a positive result indicates early detection. We compare this value to the earliest possible time gap: the difference between the first time the patient encounters the healthcare system and when they report IPV. Both the tabular model (**a**) and the fusion model (**b**) can detect IPV risk on average more than three years in advance. While the tabular model has slightly higher mean and median prediction lead times, the fusion model can detect more IPV cases in advance.

Our models can detect IPV risk years before the patients enroll in the DAIP center. In the AHC1 + DAIP1 2023 cohort, the tabular model can detect 68% of IPV cases ahead of time, while the fusion model can predict 80.6% of cases in advance. We note that the dataset only includes data from 2017 onwards to align with the rollout of a new electronic medical record system at both AHCs. This restricts our ability to identify cases at the earliest stages. We calculate a prediction-report lead time by comparing the patient’s first self-report date with the earliest date the model detected IPV; a positive result indicates early detection. Figure 3 presents the relationship between the largest possible time gap (the difference between the report date and the date with the earliest available data) and the prediction-report time gap. The tabular model achieves a mean lead time of 3.99 years and a median lead time of 5.32 years, whereas the fusion model has an average lead time of 3.68 years and a median lead time of 4.61 years. While the tabular model has higher mean and median prediction lead times, the fusion model can detect more IPV cases in advance.

### Validation results

We further validate the models on two additional validation cohorts: IPV patients enrolled in the domestic abuse intervention and prevention center at AHC2 from the same integrated care system (AHC2 + DAIP2) and AHC1 IPV patients who did not seek support from DAIP (AHC1-DAIP1). The performance of the models on the validation cohorts is shown in Fig. 2b.

For AHC2 patients, the tabular model yields an AUC of 0.81, and the clinical notes model an AUC of 0.78 while the merged model achieves an AUC of 0.82. On the AHC1-DAIP1 cohort, the tabular model yields an AUC of 0.77, and the notes model has an AUC of 0.83. The HAIM fusion model outperforms both models with an AUC of 0.84. The enrollment of IPV victims in domestic abuse intervention and prevention centers can change patterns in healthcare utilization, introducing potential feature variations when compared with the patients who disclosed IPV but did not seek help from DAIP. This could account for the variation in the tabular model’s performance. Overall, the validation results demonstrate the models’ generalizability to external populations and their potential to identify unreported IPV cases.

### Predictive features

**Fig. 4:**
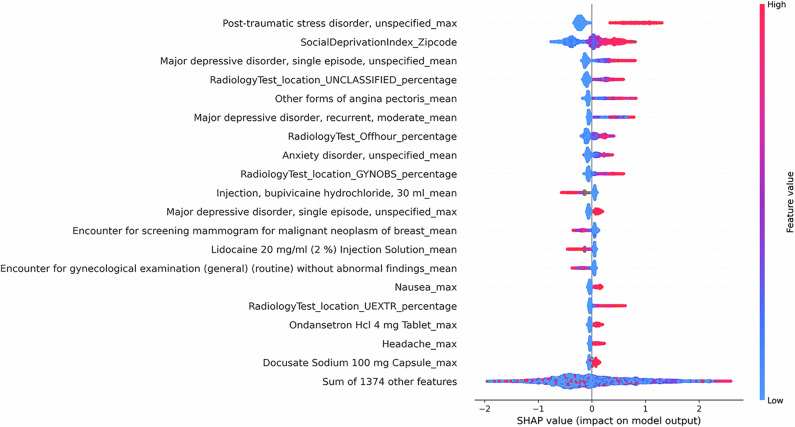
SHAP summary plot for the tabular model. The SHAP summary plots visualize the SHAP values of the 20 most important features in the tabular model. Each dot on the plot represents a prediction instance, with the color indicating the value of the feature. The position on the horizontal axis depicts the direction and magnitude contribution to the individual prediction (right for a higher likelihood of IPV, left for a lower likelihood of IPV). The SHAP summary plot gives a global understanding of feature importance and how individual features impact predictions. Features with high SHAP values for red dots indicate a potential positive correlation between high feature values and increased IPV risk, while high SHAP values for blue dots indicate the opposite.

Since the clinical texts are encoded using a transformer-based language model, it is challenging to determine which risk factors in clinical texts inform the predictions of the notes and the fusion model. Nonetheless, we can approximate the feature importance of the tabular model using the SHapley Additive exPlanations (SHAP)^29^. Using the SHAP Python library, we identify the most important features in the tabular model and use a SHAP summary plot to visualize the overall impact of these features on model predictions^30^. The SHAP summary plot is presented in Fig. 4.

We observe that specific clinical features, such as mental health disorders, chest pain, and painkiller use are correlated with a greater likelihood of IPV. Additionally, social factors such as high social deprivation are observed with higher levels of IPV risk. Aside from capturing social factors and trauma-related health outcomes, we note that the model captures injury and healthcare utilization patterns related to IPV. Notably, we observe a correlation between the high utilization of radiology tests for the upper extremity and unclassified locations (often seen in ED setting) and a higher probability of IPV presence, consistent with previous research findings^15,31^. On the contrary, high utilization of mammography and cervical cancer screening could indicate a lower likelihood of IPV presence. This observation may be explained by varied access to healthcare and preventive services across different patient populations. Patients who attend regular screening likely have better access to healthcare and support services. Additionally, their involvement in preventive healthcare services may suggest a lower fear of seeking medical attention, in contrast to the reluctance seen in IPV patients^4^.

The SHAP summary plots for language-based models (the clinical notes model and the fusion model) are provided in Supplementary Figs. S2 and S3. Since each note is represented using the language model embedding of the note, the SHAP plot shows which embedding features contribute the most to the predictions, rather than words or phrases directly from the notes. However, they provide information on which portions (type of note) contribute most to the predictions of the model, allowing the possibility to trace back to the original note when further explanation is needed.

## Discussion

This work introduces three machine learning models for the detection of intimate partner violence (IPV) in clinical settings. We developed models tailored to different data availability scenarios, including models that use only tabular data and models that incorporate clinical notes (such as radiology reports) when available. All three models demonstrate strong out-of-sample performance. Notably, the HAIM fusion model outperforms single modality models, achieving an out-of-sample area under the receiver operating characteristic curve (AUC) of over 0.8 in all cohorts. The single modality tabular model yields comparable performance, making it useful in low-resource hospitals where only limited medical data is recorded. Additionally, the tabular model can be used with interpretability frameworks like SHAP to provide insights into the risk factors of IPV, which can help clinicians better assess IPV cases and inform intervention decisions. Moreover, the models can identify risk of IPV years before patients seek help, demonstrating their potential to support proactive screening.

The HAIM fusion model performs consistently when validated on IPV patients who did not seek support from the domestic abuse intervention and prevention centers and patients from another AHC in the same network, achieving AUCs above 0.8. Even when a decline in performance is observed in either the tabular or the notes model in these validation groups, the fusion model maintains a strong performance. This result demonstrates that compared to traditional machine learning approaches that utilized a single modality of data, the multimodal modeling approach improved stability in model performance even with modality-specific feature variability when applied to an unseen group of patients. In such cases, it can be difficult to know in advance which modality’s predictions will be more reliable; by combining both, the fusion model achieves more stable performance than relying on either modality alone. In the HAIM framework, the different modalities are processed separately and only merged at the prediction stage. As a result, feature changes in one modality do not hinder learning from the other modalities. This advantage of the HAIM framework is particularly relevant in healthcare, where variations in data availability and in the recording of unstructured data (notes, images, etc.) are common across different hospitals.

The models proposed have significant clinical implications, transforming how healthcare providers identify and support patients at risk of IPV. Traditional screening methods rely only on self-reporting, often leading to underreporting due to fear, stigma, and safety concerns. A prediction tool utilizing the models proposed can address this gap by providing an objective, evidence-based risk assessment based on historical clinical data available in EMR. By enabling early identification of at-risk patients, the tool can facilitate timely, trauma-informed interventions that can prevent escalation into severe injuries, chronic health conditions, and even IPV-related homicides.

While our models demonstrate strong performance in validation cohorts, several limitations and considerations should be taken into account for their practical use. First, the model is developed using IPV patients who sought help at DAIP, which can introduce selection bias. Although we validate the models on patients who did not seek help from DAIP but carry IPV-related diagnoses, IPV victims who are reluctant to disclose their experience or seek help are underrepresented in the model, potentially limiting the models’ generalizability. Second, due to the underreported nature of IPV, the true population distribution of IPV and non-IPV patients is unknown. As a result, the distribution of patients in our validation cohorts may not accurately reflect real-world deployment settings. Careful performance evaluation on the more general population of female patients should be conducted before implementing the models for clinical use. Third, our models rely on EMR data, which may be incomplete, inconsistent, or subject to documentation biases. Lastly, while the tabular model offers an approximate understanding of predictive factors of IPV, it does not statistically attribute or quantify the impact of each factor. An important direction of future work is to conduct a rigorous statistical analysis of contributing factors. In this work, individual-level demographic variables of the patients were excluded to avoid learning a biased predictor; however, prior works have shown their value in IPV identification^18,19^. Future iterations of the model should carefully include socio-demographic variables to aid in IPV identification and evaluate their significance.

It is also important to emphasize that the models are not intended to provide a definitive diagnosis of IPV, but rather to serve as a tool to assist healthcare providers in having patient-centered conversations around IPV. By generating individualized risk probability scores, the models enable timely screening and help providers identify patients who may benefit from patient-centered, sensitive conversations and the offering of support resources, without requiring patient disclosure or assigning a formal diagnosis. Previous research has identified that intrusion and loss of autonomy during the disclosure process can hinder IPV victims’ willingness to seek help^32–34^. Therefore, when utilizing the models in any IPV screening support system, it is crucial to implement safeguards to ensure that IPV-related conversations are conducted sensitively without compromising patient autonomy.

In conclusion, this work represents the first effort towards a practical multimodal machine learning approach to IPV risk detection in healthcare settings. We plan to use our models to develop a decision support tool embedded in electronic medical record systems to provide real-time IPV risk evaluations in clinical settings. When used in a patient-centered manner, this tool can serve as a key component of a proactive approach to IPV intervention, enabling timely and effective support and ultimately leading to improved long-term health outcomes for at-risk patients.

## Methods

### Patient cohorts control demographics matching

We constructed control (non-IPV) cohorts that closely mirrored the demographic characteristics of IPV patients by optimally matching the age and demographic information of both the IPV and control patients, an approach inspired by recent work by Bertsimas et al. on mirroring randomized control trials using observational data^35^. Since the IPV group has a different demographic composition than the general patient population, this discourages the model from overfitting to demographic patterns and producing biased predictions. The initial pool of control patients provided by the hospital consists of ten randomly selected female patients within a ± 10-year age range and approximately similar racial backgrounds to each IPV patient. A logistic regression model was trained to classify patients into IPV and non-IPV groups based on demographics (race and ethnicity features). This simple approach provides an approximation of the strength of the association between each demographic variable and IPV risk via the coefficients of the logistic regression model. These coefficients are incorporated in a mathematical optimization model to encourage close matching in demographic variables that may have significantly different distributions in the IPV and control groups. Specifically, the coefficients are used to compute a weighted distance metric that quantifies the demographic similarity between each IPV and potential control patients. To achieve a closer match in age, we discretize age into 5-year age groups; each IPV patient is matched with six control patients within the same 5-year age group while minimizing the difference in their demographic features.

The final model development cohort contains 841 IPV and 5,212 control patients. Table 1 presents summary statistics of the demographics of the IPV and the optimally matched control cohort. The demographic distribution of the optimally matched control group closely mirrored that of the IPV group. Summary demographics statistics of the validation cohorts are included in Supplementary Tables 3-5.

**Table 1:** Model Development Cohort Patient Characteristics

		Total	Control	IPV
	# patients	6053	5212	841
Age	<30	16.50%	16.42%	17.00%
	30–39	30.48%	30.58%	29.85%
	40–49	23.03%	23.00%	23.19%
	50–59	15.35%	15.33%	15.46%
	60+	14.64%	14.66%	14.51%
Race	White	37.67%	38.05%	35.32%
	Black	28.65%	28.61%	28.89%
	Asian	3.65%	3.78%	2.85%
	Alaskan/Pacific	0.73%	0.58%	1.66%
	Other	20.06%	19.49%	23.54%
	Unknown	9.25%	9.50%	7.73%
	Multi-race	1.88%	1.80%	2.38%
Ethnicity	Hispanic	19.02%	19.19%	17.95%
	Non-Hispanic	71.40%	70.49%	77.05%
	Unknown	9.58%	10.32%	4.99%

Summary demographics statistics of the model development cohort, in percentages of each patient group.

### Data Sources

We consider data from the EMRs between January 2017 and December 2023. The structured data include demographics, diagnosis history, medication records, past radiology studies, past hospital visits, and vital signs (e.g., blood pressure, weight, pain scores). Since we don’t have patient-level data on social determinants of health, we approximate this with public datasets such as social deprivation and household income at the zip code level^36,37^. The dataset also contains clinical notes and reports, including radiology reports, progress notes, physical exam notes, social worker notes, and Emergency Department notes. This study was approved by the Institutional Review Board of Mass General Brigham (Protocol # 2016P002096). Informed consent was waived due to the use of retrospective data from a large cohort collected over an extended period of years. This study was conducted in accordance with the Declaration of Helsinki.

### Data preprocessing and feature engineering

In preprocessing the tabular data, since the hospital transitioned the diagnosis coding system (from ICD-9 to ICD-10) during the period considered in this study, we converted all diagnosis records in the ICD-9 to ICD-10 format. This standardization ensures consistency in representing the same diagnosis across data recorded in different periods. To prevent target leakage, we remove diagnoses that are directly indicative of IPV (e.g., adult physical/sexual abuse, encounter for mental health services for the victim and perpetrator of abuse). We include the top 5% of most frequent diagnoses and medications in the model, which results in a subset of 119 diagnoses and 187 medications. In addition, we retain specific diagnoses (e.g., head injury, diabetes) relevant to IPV based on previous research findings^2,12,13^. Additional tabular features include the frequency of emergency department (ED) visits, as prior research suggests a correlation between IPV and frequent ED visits^6^. Lastly, we extracted injury patterns, such as the injury site and time of radiology study from radiology tests. The time-related features are relevant as IPV-related visits tend to be more frequent during weekends, holidays, and outside of regular business hours^38^.

In preprocessing the clinical notes, text irrelevant to the prediction task (headers and footers) was removed. In addition, we remove sections in radiology reports that could directly contain the prediction target to prevent target leakage. For instance, the Indication/History/Reason for exam sections of the radiology report describe the clinical reason for the imaging study (e.g., assault by head strike), which can provide direct clues about the prediction task. Consequently, the model may learn to rely on this text to make predictions rather than capturing more generalized patterns that signal IPV-related injuries.

### Models

For the tabular data-only model, we leverage time-series processing techniques to aggregate and summarize the historical timestamped measurements such as diagnoses, medications, vitals, visits, etc. This involves computing statistics such as the minimum, maximum, average values, and most recent values of the measurements over the time window up to the prediction day. Meanwhile, demographic values remain static and are not subject to time-series aggregation. By capturing the temporal trends and patterns in the data, we can assess whether changes or patterns in health indicators over time indicate the presence of IPV.

For clinical notes and reports, we leverage a transformer-based bidirectional encoder model pre-trained on a large corpus of clinical texts to generate contextual embeddings of the text. The resulting fixed-length embeddings of the notes serve as inputs to the downstream models. In contrast with traditional NLP techniques such as bag-of-words or TF-IDF, this approach captures contextual nuance in the texts and the medical knowledge learned from the pre-training of the language model. Given the extended length of clinical notes and reports, we encode the text with Clinical-Longformer, a compact encoder model suitable for encoding long sequences of medical^21,39^. With the recent success of generative LLMs and their domain-specific fine-tuned variants, we also evaluate MedAlpaca 7B, a medical domain fine-tuned LLaMA 7B model^40^. We build our prediction models using both Clinical-Longformer and MedAlpaca 7B to compare their performance as contextual encoders for our task. We utilize MedAlpaca 7B for its smaller model size and extended context window, as larger models pose significant challenges in deployment. For both note-only and fusion models, MedAlpaca achieved similar performance to the Clinical Longformer with a marginal AUC difference of ± 0.02. Detailed results and comparison are presented in Supplementary Table 6. This demonstrates that a smaller model can be used without sacrificing accuracy, allowing for deployment in clinical environments with limited computational resources.

For both the tabular and text-based models, we train downstream machine learning models using either the tabular data or the embedding representations of the clinical texts. The machine learning models compared include logistic regression, decision trees, random forest, and gradient-boosted trees. In preliminary experiments, XGBoost consistently outperformed the other three methods. Therefore, we opt to use XGBoost for our final models, using the xgboost Python library^41^. We select the best hyperparameters based on Grid Search with 5-fold cross-validation on the training set and report the performance on the test set. The hyperparameter tuning is done with the GridSearchCV module in the scikit-learn Python library^42^.

**Fig. 5:**
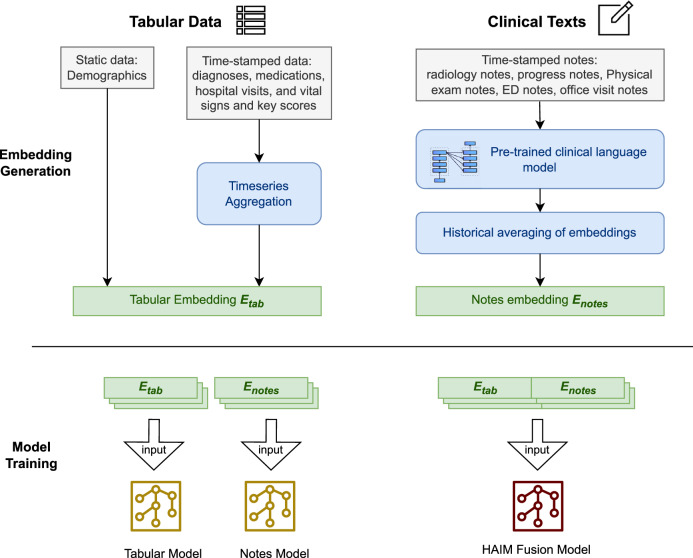
Single modality and fusion model pipeline. The tabular data includes static demographics data and time-stamped data like diagnoses, medications, and vitals. The timestamped data are processed using time-series techniques to capture historical feature variations (such as the most recent value, mean, max, etc.). The unstructured clinical texts are processed using a transformer-based clinical Large Language Model, and the resulting fixed-length vector embeddings of the clinical texts are used as inputs to the downstream models. For single-modality models, we train classification models using either the tabular data or the embedding representations of the clinical texts. The HAIM fusion model concatenates the embeddings extracted from the tabular data with those of clinical texts as inputs to the model.

The HAIM fusion model concatenates the embeddings extracted from the tabular data with those of clinical texts for model training and prediction. This concatenation enables the model to capture interactions between different modalities, such as the relationships between demographic features, clinical measurements, and textual information from the clinical texts. The pipeline of the models is outlined in Fig. 5.

## Supplementary information

Supplementary information

## Data Availability

The clinical data in this study contain protected health information (PHI), hence not publicly available. Researchers interested in collaboration should contact the corresponding authors and apply for data access through the AHC’s Institutional Review Board.

## Code availability

Due to the risk of exposing protected health information and the highly sensitive nature of IPV risk detection, the code for data analysis and modeling is not publicly available. However, details on data preparation and modeling implementation are available in the methods section. Researchers interested in more details on the methodologies can contact the corresponding author.
