# Supplementary material for: Leveraging multimodal machine learning for accurate risk identification of intimate partner violence
Source: NPJ Womens Health. 2026 Mar 13;4(1):15. doi: 10.1038/s44294-025-00126-3 (PMC12987719; doi:10.1038/s44294-025-00126-3)

Leveraging Multimodal Machine Learning for Accurate Risk Identification of Intimate Partner Violence

Jiayi Gu[1,2], Kimberly Villalobos Carballo[1,2], Yu Ma[1,2], Dimitris Bertsimas[2,3,4*], Bharti Khurana[1*‡]

[1] Trauma Imaging Research and Innovation Center, Brigham and Women's Hospital, 75 Francis St, Boston, MA 02115, USA.

[2] Operations Research Center, Massachusetts Institute of Technology (MIT), Cambridge, MA 02139, USA.

[3] Sloan School of Management, MIT, Cambridge, MA 02139, USA.

[4]Dynamic Ideas LLC, 271 Waverley Oaks Rd, Waltham, MA

* These authors contributed equally to this work

‡Corresponding author: bkhurana@bwh.harvard.edu

Supplementary Information

Supplementary Table 1A: **AUC Performance of the tabular model demographic subgroups in the validation cohorts.**

		Test set in AHC1+DAIP1 Cohort	AHC1+DAIP1 2023 Cohort	AHC2+DAIP2 2023 Cohort	AHC1-DAIP1 Cohort
Race	White	0.819	0.766	0.855	0.889
	Black	0.935	0.846	0.717	0.675
	Asian	0.864	0.652	0.534	0.571
	Unknown	0.850	0.859	0.530	0.823
	Other	0.812	0.868	0.725	0.778
Ethnic Group	Hispanic	0.902	0.841	0.631	0.794
	Non-Hispanic	0.845	0.826	0.848	0.766
	Unknown	0.923	0.847	0.473	0.827

Supplementary Table 1B: **AUC Performance of the fusion model demographic subgroups in the validation cohorts.**

		Test set in AHC1+DAIP1 Cohort	AHC1+DAIP1 2023 Cohort	AHC2+DAIP2 2023 Cohort	AHC1-DAIP1 Cohort
Race	White	0.868	0.850	0.857	0.908
	Black	0.942	0.916	0.702	0.786
	Asian	0.808	0.637	0.704	0.681
	Unknown	0.908	0.874	0.725	0.883
	Other	0.818	0.912	0.726	0.889
Ethnic Group	Hispanic	0.926	0.857	0.637	0.899
	Non-Hispanic	0.870	0.885	0.840	0.828
	Unknown	0.938	0.909	0.548	0.855

Supplementary Table 2 Accuracy, sensitivity, and specificity of the Tabular and Fusion models on the AHC1+DAIP1 cohorts. Bold highlights the chosen probability thresholds.

		Test set in AHC1+DAIP1 2017-2022 cohort (N=6,053)					AHC1+DAIP1 2023 cohort (N=1,556)				
	Threshold	Accuracy	Sensitivity	Specificity	PPV	NPV	Accuracy	Sensitivity	Specificity	PPV	NPV
Tabular Model	**0.4**	79.1%	**73.6%**	**82.6%**	**73.6%**	**82.6%**	**73.5%**	**73.4%**	**73.5%**	**64.4%**	**80.9%**
	0.5	80.3%	67.6%	88.6%	79.7%	80.6%	74.0%	61.9%	81.9%	76.7%	74.0%
	0.6	80.2%	67.0%	88.9%	76.8%	81.5%	74.1%	60.5%	82.9%	76.8%	81.5%
Fusion Model	**0.4**	**79.1%**	**87.5%**	**73.7%**	**68.7%**	**89.9%**	**76.9%**	**86.9%**	**70.3%**	**65.7%**	**81.2%**
	0.5	79.8%	70.5%	85.9%	76.8%	81.2%	79.8%	66.1%	88.7%	79.3%	80.1%
	0.6	79.8%	70.3%	86.0%	76.9%	81.5%	79.9%	55.8%	89.1%	79.8%	80.0%

Supplementary Table 3: Summary demographics statistics of the AHC1+DAIP1 2023 cohort, in percentages of each patient group.

		Total	Control	IPV
	# patients	1,556	1,333	223
Age	<30	24.74%	24.53%	26.01%
	30-39	33.55%	33.68%	32.74%
	40-49	19.79%	19.80%	19.73%
	50-59	14.72%	14.78%	14.35%
	60+	7.20%	7.20%	7.17%
Race	White	31.36%	32.18%	26.46%
	Black	32.01%	32.26%	30.49%
	Asian	3.28%	3.30%	3.14%
	Alaskan/Pacific	0.32%	0.30%	0.45%
	Other	21.47%	19.35%	34.08%
	Unknown	11.57%	12.60%	5.38%
	Multi-race	1.86%	1.80%	2.24%
Ethnicity	Hispanic	37.53%	39.53%	25.56%
	Non-Hispanic	43.77%	39.23%	70.85%
	Unknown	18.70%	21.23%	3.59%

Supplementary Table 4: Summary demographics statistics of the AHC2+DAIP2 cohort, in percentages of each patient group.

		Total	Control	IPV
	# patients	1,316	1,130	186
Age	<30	27.89%	27.79%	28.49%
	30-39	28.65%	28.76%	27.96%
	40-49	21.20%	21.15%	21.51%
	50-59	14.36%	14.34%	14.52%
	60+	7.90%	7.96%	7.53%
Race	White	53.88%	54.25%	51.61%
	Black	13.75%	13.36%	16.13%
	Asian	6.16%	6.28%	5.38%
	Alaskan/Pacific	0.91%	0.71%	2.15%
	Other	13.37%	12.21%	20.43%
	Unknown	11.93%	13.19%	4.30%
	Multi-race	2.43%	2.12%	4.30%
Ethnicity	Hispanic	6.91%	7.43%	3.76%
	Non-Hispanic	72.49%	70.44%	84.95%
	Unknown	20.59%	22.12%	11.29%

Supplementary Table 5: Summary demographics statistics of the AHC1-DAIP1 cohort, in percentages of each patient group.

		Total	Control	IPV
	# patients	664	569	95
Age	<30	30.27%	30.23%	30.53%
	30-39	26.36%	26.36%	26.32%
	40-49	15.96%	15.99%	15.79%
	50-59	9.49%	9.49%	9.47%
	60+	17.92%	17.93%	17.89%
Race	White	28.01%	27.94%	28.42%
	Black	39.61%	40.60%	33.68%
	Asian	2.86%	2.64%	4.21%
	Alaskan/Pacific	0.30%	0.18%	1.05%
	Other	25.30%	24.96%	27.37%
	Unknown	3.92%	3.69%	5.26%
	Multi-race	3.92%	4.22%	2.11%
Ethnicity	Hispanic	24.40%	25.66%	16.84%
	Non-Hispanic	70.78%	69.42%	78.95%
	Unknown	4.82%	4.92%	4.21%

Supplementary Table 6: AUC performance comparison of the Notes Model and Fusion Model with Clinical-Longformer and MedAlpaca 7B.

Cohort	Notes Model (Clinical-Longformer)	Notes Model (MedAlpaca 7B)	Fusion Model (Clinical-Longformer)	Fusion Model (MedAlpaca 7B)
Test set in AHC1+DAIP1 2017–2022 cohort	0.869	0.874	0.875	0.883
AHC1+DAIP1 2023 cohort	0.877	0.885	0.880	0.894
AHC2+DAIP2 2023 cohort	0.779	0.743	0.816	0.814
AHC1–DAIP1 cohort	0.831	0.860	0.842	0.865

Supplementary Figure 1: Calibration curves of the Tabular Model (**a**) and the Fusion Model (**b**). The predicted probabilities from both models are calibrated using isotonic regression. The calibrated probabilities show good concordance with observed probabilities of IPV risk.

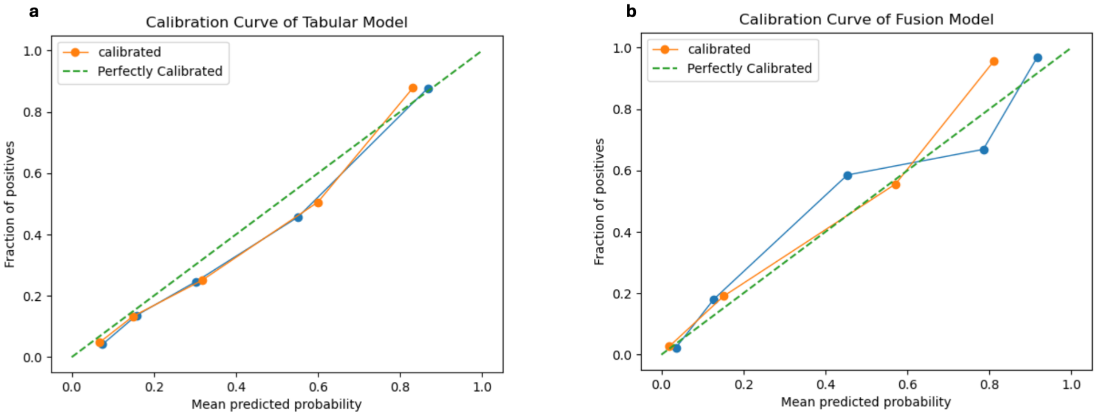

Supplementary Figure 2: The SHAP summary plot for the clinical notes-only model. In the notes model, each note is represented using the language model embedding of the note. Therefore, the features on the y-axis represent embedding dimensions that contribute the most to predictions rather than interpretable words or phrases. Nevertheless, it provides insights into which type of clinical notes are the most significant when detecting the risk of IPV.

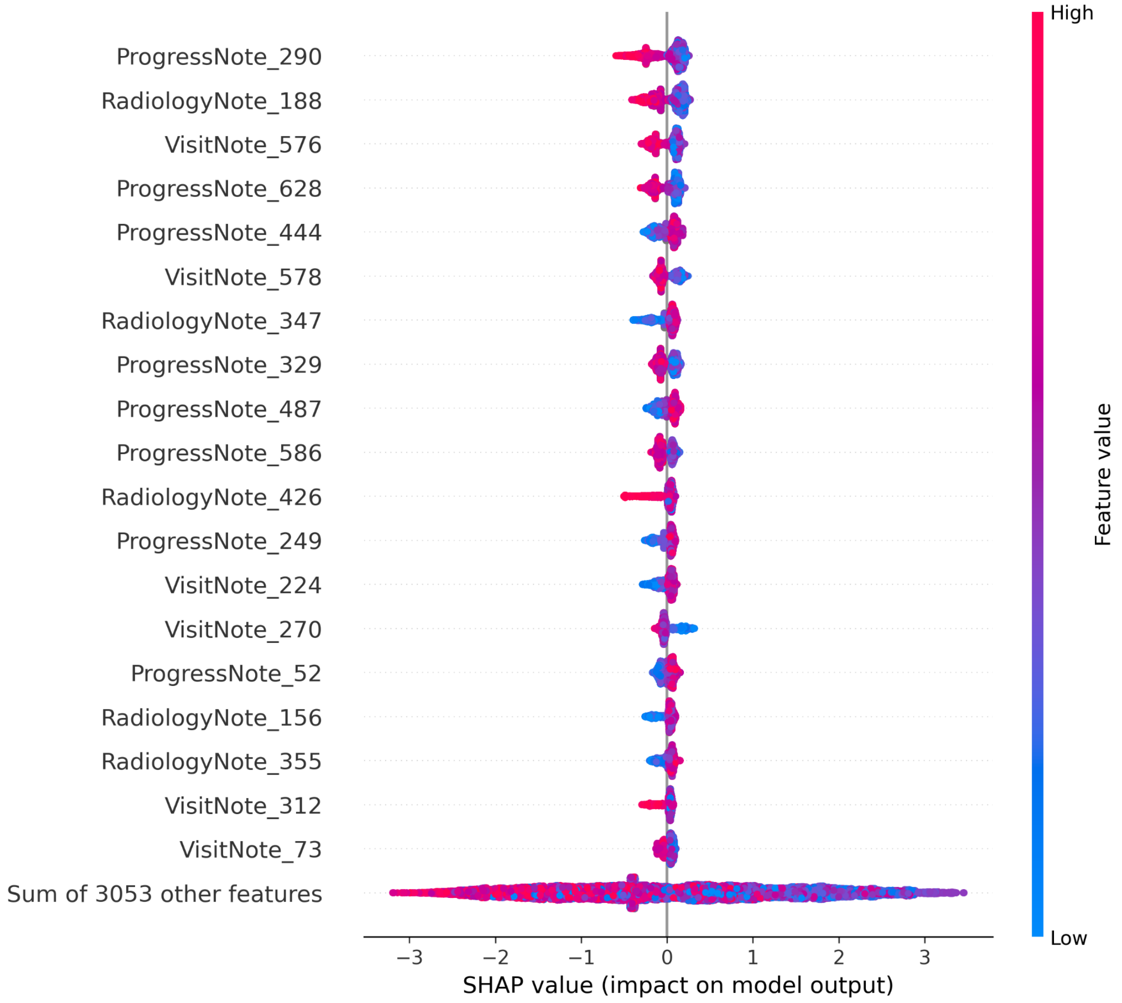

Supplementary Figure 3: The SHAP summary plot for the HAIM fusion model. With the use of language model embedding representations of the notes, the SHAP plot shows which language embedding features contribute the most to the predictions, rather than words or phrases. Nevertheless, it provides insights on which type of clinical notes are the most significant when detecting risk of IPV, and their relative contributions compared to the tabular features.

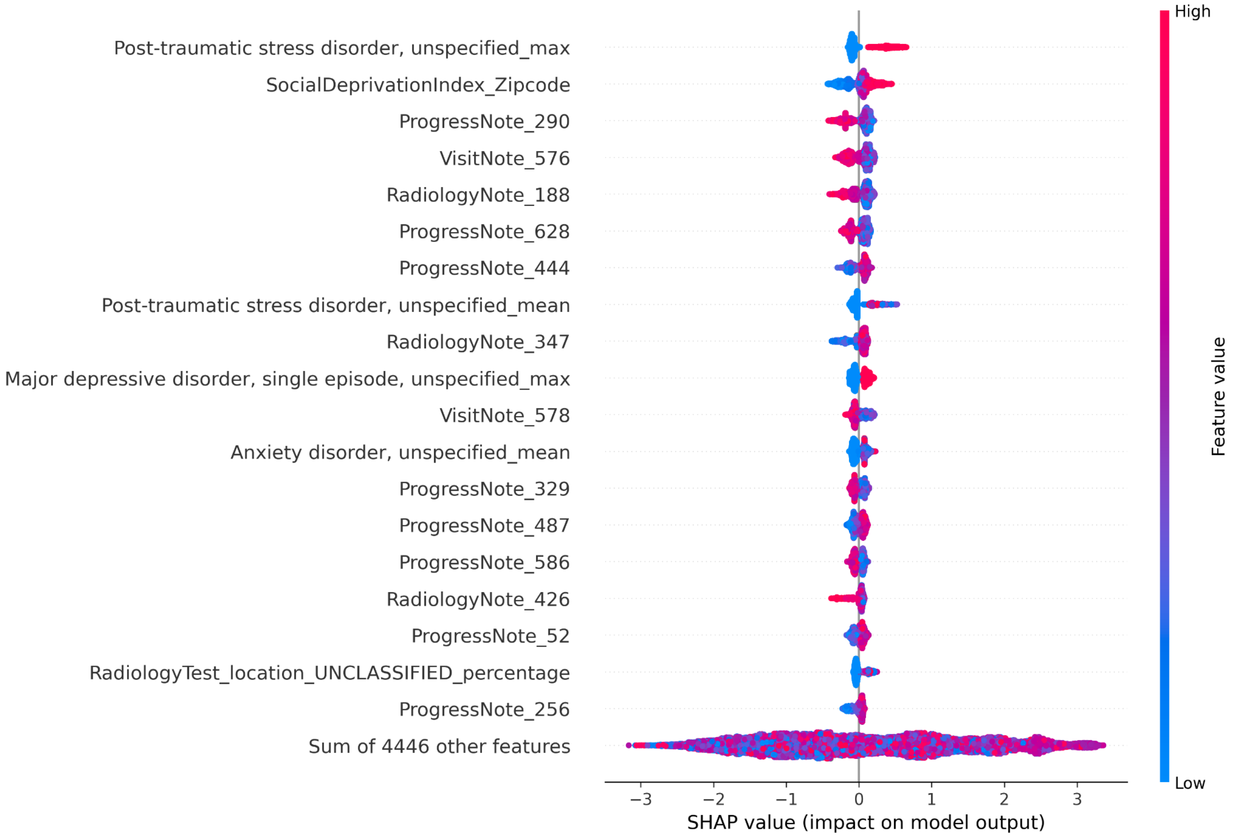

Supplement: Supplementary file 1 — Supplementary information [file 44294_2025_126_MOESM1_ESM.pdf]
